# Protocol for stable cell line production to express muscle-type nicotinic receptor

**DOI:** 10.1016/j.xpro.2026.104427

**Published:** 2026-03-11

**Authors:** Anna Li, David B. Sauer, Yin Yao Dong

**Affiliations:** 1 Centre for Medicines Discovery, Nuffield Department of Medicine, University of Oxford, Oxford OX3 7FZ, UK; 2 Nuffield Department of Clinical Neurosciences, University of Oxford, Oxford OX3 9DS, UK

**Keywords:** Cell culture, Cell isolation, Protein expression and purification

## Abstract

The adult muscle-type nicotinic acetylcholine receptor (AChR) is essential for neuromuscular transmission but is difficult to produce due to the requirement for coordinated subunit assembly. Here, we present a protocol for generating doxycycline-inducible stable cell lines that co-express all four subunits. We describe steps for lentivirus infection, puromycin selection, fluorescence-activated cell sorting, clonal expansion, and protein expression test. This protocol enables AChR production at quantities sufficient for single-particle cryoelectron microscopy (cryo-EM) and may be applicable to other hetero-multimeric protein complexes.

For complete details on the use and execution of this protocol, please refer to Li et al.^1^

## Highlights

• Guidance on designing lentivirus constructs to express human muscle-type AChR subunits
• Steps for lentiviral co-infection and antibiotic selection for stably integrated cells
• Instructions for FACS isolation of clones expressing all AChR subunits
• Procedures for AChR expression test by ^125^I-α-bungarotoxin binding assay

## Abstract (editor highlights)

Publisher’s note: Undertaking any experimental protocol requires adherence to local institutional guidelines for laboratory safety and ethics.

## Graphical abstract

**Figure:**
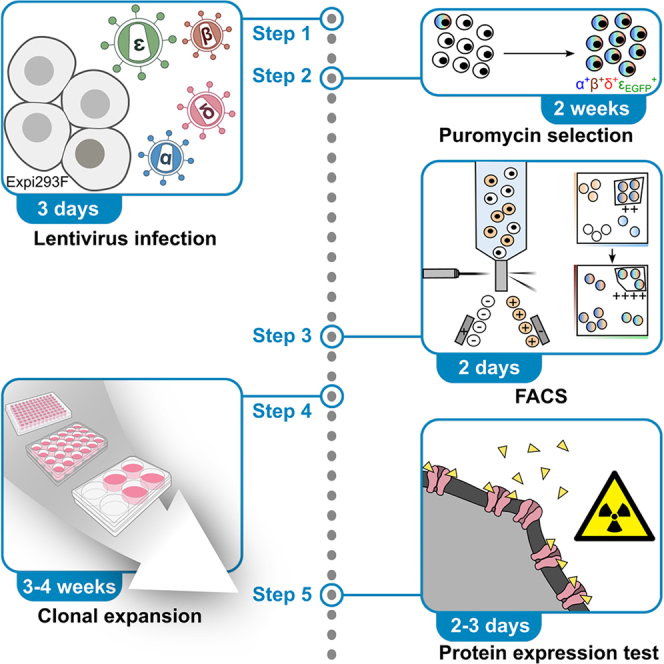

## Before you begin

### Introduction to AChR structure and function

The muscle-type nicotinic acetylcholine receptor shares the same structural fold as other pentameric ligand-gated ion channels (pLGICs) such as GABA receptors, glycine receptors, 5HT3 receptors, and the zinc-activated channel.^2^ In the case of muscle-type AChR, the channel pentamer is formed of five subunits: α, β, δ, and ε. Each of the subunits consist of an extracellular domain containing ten β strands arranged into a β sandwich, a pore domain with four transmembrane helices, a juxtamembrane helix lying parallel to the inner leaflet of the plasma membrane, and an intracellular domain that is largely disordered.^1^ Binding of two acetylcholine molecules to the extracellular domain of muscle-type AChR activates the receptor through a series of structural changes in the protein, ultimately opening the channel’s hydrophobic gate and thus allowing passage of hydrated cations. This AChR activation depolarizes the sarcolemma in response to signaling from the motor neuron terminal, thereby initiating skeletal muscle contraction. Mutations in AChR subunits account for ∼50% of cases of congenital myasthenic syndrome (CMS), a rare neuromuscular disorder characterized by fatigable weakness.^3^ Additionally, AChR-directed autoantibodies are detected in approximately 80% of patients with myasthenia gravis, an autoimmune disease affecting approximately 1 in 10,000 people.^4^ Therefore, to enable structural, biochemical, and target engagement studies critical for understanding disease mechanisms and enabling AChR targeting by new therapeutics, we have developed a method for the recombinant production of biochemically pure receptor.

### Background of stable cell line generation for pLGIC expression

Stable cell lines have numerous advantages for expression of target protein, with particular advantages over other methods in cost, reproducibility, and production of protein complexes. In the study of the muscle-type nicotinic acetylcholine receptor, this was first demonstrated with the generation of the DB40 cell lines from the human rhabdomyosarcoma cell line TE671 in the 1990s for modelling neuromuscular diseases via increased expression of the adult AChR (αβδε).^5^^,^^6^ The fetal AChR (αβδγ) expressing TE671 rhabdomyosarcoma cells were sequentially transfected with plasmid cDNAs encoding the AChR-ε, β, δ subunits. Between each transfection, the clones were subjected to limited dilution or antibiotic selection, followed by ^125^I-α-bungarotoxin (αBuTx) radioligand binding assays to confirm increased surface AChR expression after each round of transfection. While ultimately successful in producing a cell line with enhanced expression of adult AChR (αβδε), this sequential approach requires significant manual effort and is very time consuming.

While this early work in stable AChR expression used constitutive promoters to drive protein production, more recently the tetracycline-inducible (Tet-On) promotor^7^ has been used for controlled expression of pentameric ligand-gated ion channels such as the heteromeric α1β3γ2 GABA_A_ receptor and the homomeric 5-HT3A receptor.^8^^,^^9^^,^^10^^,^^11^ When the doxycycline-inducible expression is combined with lentiviral gene delivery, the yield of homomeric β3 GABA_A_ receptor is five-fold higher compared to transient transduction with BacMam.^12^ This technology has been commercialized in kits. For example, the Lenti-X Tet-One system (Takara) comes with lentiviral packaging and gene delivery under an inducible promoter. Alternatively, the Flp-In T-REx 293 system (Invitrogen) inserts a gene of interest into a host cell’s established recombination site with an inducible promoter. For comparison of site-specific recombination (Flp-In, Jump-In), transposase-mediated integration (Sleeping Beauty, PiggyBac), and lentiviral gene delivery, see paper by Fong and Ceroni.^13^ Although stable cell line expression has significant advantages over introducing the AChR genes via transient transfection or BacMam systems (Table 1), the commercial kits for generating these cell lines can be expensive and inflexible to the experimental design. Furthermore, these kits are poorly suited to the production of multi-protein complexes, such as AChR, which require the stable incorporation and expression of multiple genes.

**Table 1:** Choosing gene delivery and expression systems for recombinant AChR production in mammalian cells

	BacMam	Lentivirus	Transient transfection
Required resources	BacMam expression vector e.g., pHTBV *(MultiBacMam)*^a^ biGMamAct^14^	Gene transfer vector e.g., TLCV2 *(Lenti-X system from Takara Bio)*^a^ *(T-REx system from ThermoFisher)*^a^	Mammalian expression vector e.g., pcDNA3.1 Transfection reagent e.g., polyethylenimine (PEI)
Has this yielded heterooligomeric membrane protein structures?	Yes^15^^,^^16^^,^^17^^,^^18^	No,but protein expression demonstrated for GABA_A_R^12^	Yes^19^^,^^20^^,^^21^^,^^22^
Expected maximum expression level	High	Medium-high, but susceptible to epigenetic silencing	Low-medium
Exceptional Safety requirements beyond standard cell culture	–	Institutional and regulatory requirements for lentivirus and GMO generation	–
Protein expression cost	Medium	Medium	High, due to higher consumables and cell culture costs for plasmid DNA amplification and purification and transfection reagents
Expected time from successful cloning to protein production	>3 weeks per subunit	>3 months	1–2 week

a Kits in parenthesis e.g., MultiBacMam, Lenti-X and T-REx are available for purchase.

### Innovation

To address the limitations of existing techniques, we developed a protocol for generating monoclonal stable cell lines without the need of commercial kits whilst still retaining the benefits of both doxycycline induction and lentiviral transduction. We demonstrate the reproducible and robust expression of adult muscle-type AChR, using co-expression of different fluorescent proteins to ensure production of the heteromeric protein complex. Once construct design is complete, progress can be fast-tracked by outsourcing gene synthesis and lentivirus packaging to commercial services with turnaround times of as little as one month. This means the experimenter needs only to perform the hands-on steps starting from lentiviral infection to protein expression testing.

### Cell culture

Expi293F cells (Gibco) were purchased from Thermo Fisher Scientific for this study. Cells were maintained in Freestyle 293 medium supplemented with 0.1% v/v penicillin-streptomycin-amphotericin B mix (PSA, Lonza) or 0.1% v/v penicillin-streptomycin (PS, Invitrogen or Merck). During the prolonged incubation time in the early stages of clonal expansion, a higher concentration of 1% v/v PSA was added to prevent contamination. This will be referred to as **growth medium**. All buffers and reagents should be prepared in advance and sterilized using a 0.22 μm filter.

For adherent cells, standard culture conditions are 37°C, 5% CO_2_, in a humidified stationary incubator. For suspension cells, standard culture conditions are 37°C, 8% CO_2,_ 100–250 rpm, and 75% humidity. We used an Infors HT Multitron Cell shaking incubator with a 25 mm throw. Shaking speed varied depending on the cell culture vessel. For example, 24-well blocks (VWR) were incubated at 250 rpm, 100–500 mL vented Erlenmeyer flasks (Corning or Jet Biofil) were incubated at 100–150 rpm, and roller bottles (Greiner Bio-One) were incubated at 150–180 rpm. For the same type of vessel, we increased the speed with higher volume within the stated range. Cells should be maintained at a density of 0.3–3×10^6^ cells/mL. For protein expression, cells were seeded at 1×10^6^ cells/mL and induced by adding doxycycline the following day.

### Construct design

Complementary DNAs (cDNA) encoding human wildtype AChR α1- (P3A negative isoform), β1-, δ-, and ε-subunits in pcDNA3.1-hygro were provided by Professor David Beeson.^23^ An additional EGFP-tagged construct of the ε subunit (εEGFP) was previously generated by inserting EGFP from pEGFP-N1 (BD Biosciences) into the M3-M4 loop of the wildtype ε sequence.^24^

**Figure 1:**
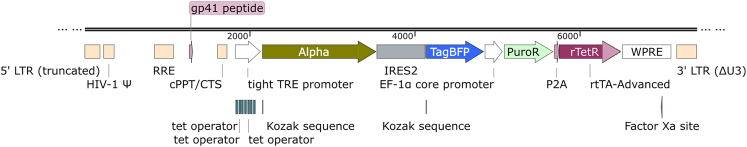
Transfer vector design for co-expression of AChR α and tagBFP Transfer vector design for β and δ subunits were constructed similarly with mIFP, mCherry, and EGFP inserted downstream of IRES2 instead of tagBFP. LTR, long terminal repeat from HIV-1; HIV-1 Ψ, packaging signal; RRE, Rev response element of HIV-1; cPPT/CTS, central polypurine tract and central termination sequence of HIV-1; TRE, tetracycline response element; IRES2, internal ribosome entry site 2; puroR, puromycin N-acetyltransferase; P2A, 2A peptide from porcine teschovirus-1; rtTA-Advanced, improved tetracycline-controlled transactivator; WPRE, woodchuck hepatitis virus posttranscriptional regulatory element.

**Figure 2:**
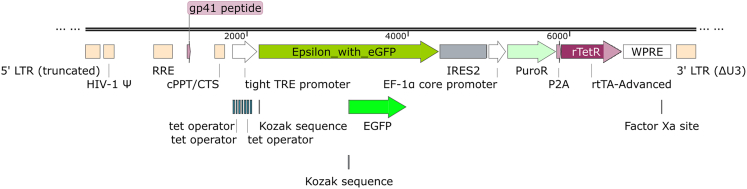
Transfer vector design for the expression of AChR ε_EGFP_ fusion protein Vector element abbreviations are the same as Figure 1.

Lentivirus cloning was completed by the Genome Engineering Facility at the Weatherall Institute of Molecular Medicine. The doxycycline-inducible lentiviral vector was generated from the TLCV2 vector (AddGene: 87360) by removing the Cas9 sequence from the original vector, inserting a KOZAK sequence (CCACC), and an internal ribosome entry site (IRES2) followed by fluorescent protein cDNA of tagBFP, mCherry or mIFP. Subsequently, the α, β, and δ subunit cDNAs were cloned into the lentivirus vector after the KOZAK motif, and upstream of the IRES2 and fluorescent protein sequences, to create the following co-expression constructs: α-tagBFP, β-mCherry, β-mIFP, and δ-mCherry (Figure 1).^25^^,^^26^^,^^27^^,^^28^ Using this method, the gene of interest is separated from a downstream fluorescent protein marker by an IRES2 sequence, and both are controlled by the doxycycline inducible TRE promotor. We also considered replacing the IRES2 sequence with the P2A sequence from porcine teschovirus-1 2A, which would also allow co-expression of AChR subunits and fluorescent proteins. However, the P2A sequence would add a non-native Asn-Pro-Gly sequence to the target channels with unknown effects, and therefore we selected the scarless IRES2 co-expression method.^29^ As the εEGFP construct already contained a fused, fluorescent protein, no fluorescent protein was included following the IRES2 sequence (Figure 2). Final constructs were verified by Sanger sequencing.

### Lentivirus packaging

Lentiviral particles were produced by transfecting the gene delivery plasmid, along with lentiviral packaging and envelope plasmids (pHR_SIN, pCMV delta8.91/psPAX2, pMDG/pMD2.G) into a mammalian cell line.^30^ Lentivirus-containing cell culture supernatant was harvested and titrated before being received and stored at −80°C. Viruses used in this study were produced by a core facility within the University of Oxford, generated from in-house genes and vectors, although commercial providers offer end-to-end services for custom gene synthesis and ready-to-use lentivirus.

**Figure 3:**
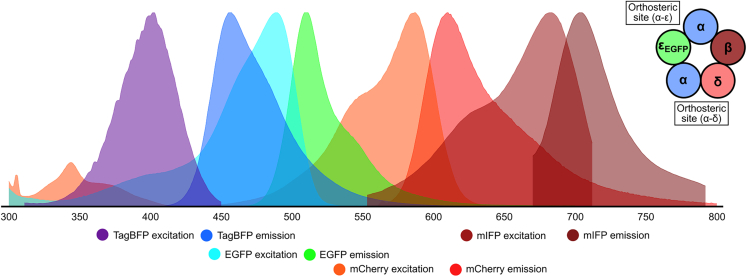
Fluorescent markers for FACS The 4-colour system consists of α_tagGFP, β_mIFP, δ_mCherry and ε_EGFP_ fusion, such that each protein is co-expressed with a different fluorescent protein. Fluorescence spectra were generated by FPbase.^31^

To select for cells expressing all four AChR subunits by fluorescence-activated cell sorting (FACS), we elected to co-express the genes with unique fluorescent protein markers (Figure 3).

### Institutional permissions (if applicable)

#### Sterile cell culture

This protocol has several steps that are at high risk of contamination, particularly the long colony expansion stage that involves prolonged culturing and repeated passaging. To minimize the risk of contamination, all cell culture was carried out in a sterile and regularly cleaned Biosafety level 2 hood designated exclusively for mammalian cell culture. This was within a cell culture facility that excludes fungal or bacterial cultures, isolated from the general lab space, and has separate lab coats and equipment.

Good sterile technique included wiping down all objects going into the tissue culture hood with sterilizing liquid such as 70% ethanol and ensuring all glassware and plasticware are sterilized. The exterior packaging of pre-sterilized equipment and any material temporarily removed from the cell culture hood, such as tubes to be centrifuged, was wiped down before placing in the hood.

#### Radiation safety

If proceeding with the ^125^I-α-bungarotoxin binding assay, ensure a risk assessment is approved and all safety procedures are followed for work with radioactive material. This includes risk assessments for shipping, storing, and disposal of the radioactive substance as well as performing the steps for this specific assay following institutional and national guidelines. The work should only be carried out in a designated and licensed space for studies with unsealed ^125^I radioactive sources, with appropriate safety and monitoring equipment for this gamma and x-ray source.

#### Working with lentivirus

Depending on institutional and regulatory requirements, biosafety approval may be required before beginning work with lentivirus. Similarly, the stable cell lines may require advanced approval for a genetically modified organism. This study required a risk assessment for the use of replication-impaired lentiviral vectors and lentiviruses. The risk assessment was made according to the UK Genetically Modified Organisms (Contained Use) Regulations 2014 guidance, which was first approved by the departmental safety officer, then by the University Biology Safety Officer of the University of Oxford, before final review and approval by the UK Health and Safety Executive (HSE). The risk assessment noted that different stages of this protocol would be carried out at differing containment levels depending upon the risk level: i) *E*. *coli* work at UK containment level 1 (BSL-1), ii) lentiviral work at UK containment level 2 in combination with a Class II microbiological safety cabinet (BSL-2), and iii) work with modified human cell lines at UK containment level 2 (BSL-2).

### Thawing of Expi293F cells

**Timing: 2 weeks**

1. (Day 0) Prepare **growth medium** by adding 0.1–1% v/v penicillin-streptomycin (PS) to Freestyle 293 medium that’s pre-warmed to 37°C in a water bath.
2. Aliquot 50 mL growth medium to a 250 mL or 500 mL vented plastic Erlenmeyer flask.

***Note:*** If the tissue culture lab has frequent fungal contamination, the use of penicillin-streptomycin-amphotericin B (PSA) is recommended over penicillin-streptomycin only.

3. Transport a frozen tube of Expi293F cells to the tissue culture lab on dry ice.
4. Rapidly thaw the cells in a 37°C water bath until the ice is reduced to the size of a grain of rice and immediately add to the flask of growth medium.

**CRITICAL:** The thawing step should be done quickly to avoid cell death. It should only be performed when all other materials are ready, such as pre-warmed and aliquoted growth medium.

***Optional:*** The cells can be washed briefly to remove DMSO prior to outgrowth. Immediately after thawing, centrifuge the cells at 100–200 *g* at room temperature for 3–5 min, carefully remove the cell freezing media without disturbing the cell pellet, then resuspend the cells in fresh growth media for transfer to the flask.

5. Incubate the cells at 100 rpm, 37°C, 75% humidity and 8% CO_2_.
6. Each day, measure cell viability and density with an automated cell counter (Invitrogen) after mixing 10 μL cells with 10 μL of 0.4% trypan blue stain (Invitrogen). Alternatively, this can be counted manually with a hemocytometer.

***Note:*** Cell viability may be low initially, but it should recover to >90% over the first week.

***Note:*** Passage the cells when the density is approximately 2×10^6^ cells/mL, typically 3–4 days post-thaw.

7. To passage the cells, the suspension culture is diluted with pre-warmed growth medium to maintain a density of 0.3–3×10^6^ cells/mL.

***Note:*** It is recommended to freeze many aliquots of low passage number Expi293F cells for future use.

8. To freeze cells, growth-phase cell culture at a density of ∼1–2.5×10^6^ cells/mL were centrifuged at 100–300 *g* for 3–5 min.
  a. Aspirate off the medium and gently resuspend the cell pellet with a serological pipette in growth medium supplemented with 5–10% sterile DMSO to final concentration of > 1×10^6^cells/mL.
  b. Aliquot 1 mL of cell suspension per cryogenic tube, label with alcohol-resistant marker, and place into a prepared cryopreservation container rated for 1°C per minute cooling, such as Mr. Frosty (Thermo Fisher) or CoolCell (Corning).
  c. After loading the cryopreservation container at room temperature, transfer it to a −80°C freezer overnight.
  d. For long-term storage (>3–6 months), frozen cells should be transferred to liquid nitrogen storage tanks.

### Puromycin kill curve to optimize selection

**Timing: 2 weeks**

9. Day −1: Dilute growth phase Expi293F to 1×10^6^ cells/mL, seed 3 mL cells per well in a 24-well block, cover with porous adhesive film (VWR) and incubate overnight at 37°C, 230–250 rpm, 75% humidity and 8% CO_2_.
10. Day 0: Cell density should have reached 1.5–2×10^6^ cells/mL. To each well, add puromycin (Merck) to a final concentration of 0–100 μg/mL. Continue to culture cells in the shaking incubator.
11. Day 1: Measure the percentage cell viability with an automated cell counter (Invitrogen) or a hemocytometer.

**CRITICAL:** If using an automated cell counter, it is important to confirm the viability measurement by eye under a light microscope. Sometimes large cell debris may be detected as live cells when all cells had in fact been killed by puromycin.

12. Repeat the sampling every 1–3 days until full elimination of Expi293F cells are observed at the higher puromycin concentrations. Identify the selection concentration using the minimum time and puromycin concentrations sufficient to kill all Expi293F cells.

**Figure 4:**
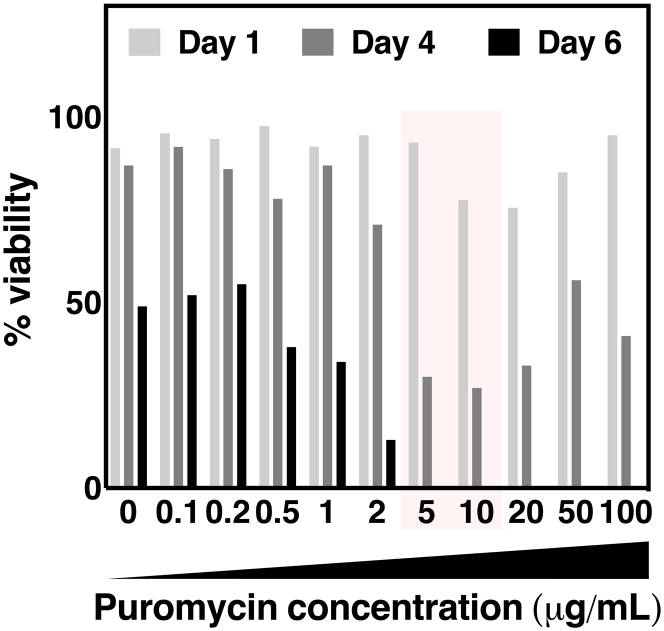
Example puromycin kill curve Puromycin at 0–100 μg/mL was added to 3 mL of 1.5×10^6^/mL growth phase Expi293F cells and viability measured by a cell counter.

***Note:*** For example, we tested puromycin at 0, 0.1, 0.2, 0.5, 1, 2, 5, 10, 20, 50 and 100 μg/mL, and sampled the cell culture on days 1, 4 and 6. All samples contained live cells on days 1 and 4, however, by day 6, cultures with ≥5 μg/mL puromycin were fully eliminated (Figure 4). Therefore, the minimum conditions for selection would be 6-day incubation at 5–10 μg/mL puromycin.

## Key resources table

**Table:** 

REAGENT or RESOURCE	SOURCE	IDENTIFIER
**Chemicals, peptides, and recombinant proteins**		

Freestyle 293 medium	Gibco	Cat# 12338018
Penicillin-streptomycin	Merck	Cat# P0781
Penicillin-streptomycin	Gibco	Cat# 15140122
Penicillin-Streptomycin-Amphotericin B mix	Lonza	Cat# 17-745E
Polybrene reagent (hexadimethrine bromide)	Merck	Cat# TR-1003-G
Puromycin	Merck	Cat# P8833
Trypan Blue Solution, 0.4%	Gibco	Cat# 15250061
Doxycycline	Merck	Cat# D3072
Trypsin-EDTA	Gibco	Cat# 15400054
Sodium butyrate	Merck	Cat# 8451440100
EDTA-free protease inhibitor cocktail	Roche	Cat# 04693132001
^125^I-α-bungarotoxin	Revvity	Cat# NEX126050UC
Phosphate buffered saline (PBS)	Oxoid	Cat# BR0014G

**Experimental models: cell lines**		

Expi293F	Gibco	Cat# A14527

**Recombinant DNA**		

TLCV2	Barger et al.^32^	Addgene 87360
pCMV delta8.91 (p8.91)	Susac et al.^33^	Addgene 187441
psPAX2	psPAX2 was a gift from Didier Trono	Addgene 12260
pMDG	Susac et al.^33^	Addgene 187440
pMD2.G	pMD2.G was a gift from Didier Trono	Addgene 12259
pcDNA3.1-hygro-CHRNA1 (P3A negative isoform)	Cetin et al.^23^	
pcDNA3.1-hygro-CHRNB1	Cetin et al.^23^	
pcDNA3.1-hygro-CHRND	Cetin et al.^23^	
pcDNA3.1-hygro-CHRNE-EGFP	Ealing et al.^24^	
EGFP	Cormack et al.^25^	
mCherry	Shaner et al.^26^	
TagBFP	Subach et al.^27^	
mIFP	Yu et al.^28^	
pEGFP-N1	Clontech	N/A

**Software and algorithms**		

SnapGene version 8.2.1	www.snapgene.com	

**Other**		

BD FACSAria Fusion Flow Cytometer	BD Biosciences	N/A
Cobra-II Auto Gamma Counter	Packard	N/A
Multitron Cell shaking incubator	Infors HT	N/A
Nunc cryogenic tubes	Thermo Scientific	363401
96-well round bottom plate	Corning	3799
48-well plate	Corning	3548
24-well plate	Corning	3526
12-well plate	Corning	3513
6-well plate	Corning	3516
24-well deep-well block	VWR	734–1217
Porous adhesive film	VWR	391–1251
Countess automated cell counter	Invitrogen	N/A
Chemgene disinfectant	MEDLAB	SKU075A or SKU074A
Vented Erlenmeyer flask, 500 mL	Jet Biofil	JBE500
Vented Erlenmeyer flask, 500 mL	Corning	431145
FACS tube	BD Falcon	352054
Count-Off surface cleaner	Revvity	6NE942T
Decon 90	Decon Laboratories Ltd	N/A

## Materials and equipment

### FACS settings

• Equipment: BD FACSAria Fusion Flow Cytometer (inside a class II Biological Safety Cabinet).
• Nozzle size: 100 μm.
• Collection: one cell per well into 96-well TC plate.
• Fluorescent channels: see Table 2.

**Table 2:** Fluorochromes and corresponding channels (excitation wavelength-emission filter in nm)

TagBFP	405–450/50 i.e., DAPI, PB, BV421
EGFP	488–525/50 i.e., FITC, GFP, Alexa Fluor 488
mCherry	561–610/20 i.e., PE-TxRd, PE-ef594
mIFP	640–710/50 i.e., Alexa Fluor 700

Useful information for instrument booking (estimated from prior experience, not essential):

• Approximate sample size (e.g., 4.5×10^6^ cells in an Eppendorf tube).
• Frequency of target cell population (e.g., 1.4% for quadruple positive cells).
• Required number of sorted cells (e.g., 18x 96-well TC plates).

Notes:

• The make and model of cell sorter is not so important as long as it can be configured for the appropriate fluorophores and nozzle size.
• Cell sorting creates aerosols. The equipment set-up must comply with local biosafety regulations, for example, a cell sorter placed inside a class II Biological Safety Cabinet is required for lentivirus-containing samples. For this reason, many FACS facilities require prior risk assessment before the experiment takes place.

## Step-by-step method details

### Infecting Expi293F cells with lentivirus

**Timing: 3 days**

1. Day 0: In a vented Erlenmeyer flask, seed 50 mL of Expi293F cells at 1×10^6^ cells/mL density, and culture at 37°C, 5% CO_2_ overnight.
2. Day 1: Spin down cells at 100–200 *g* for 3–5 min, resuspend to 0.33×10^6^ cells/mL density in growth medium supplemented with 10 μg/mL polybrene.

***Note:*** Polybrene increases infection efficiency of lentivirus by neutralizing charge repulsion between the virus and host cell membranes.

3. For each sample, aliquot 3 mL (1×10^6^) cells per well in a 24-well block.
4. Mix virus according to the pre-determined titre to achieve α:β:δ:ε_EGFP_=1:1:1:1 (Table 3). Include both multiplicity of infection (MOI)=1 and MOI=0.5 (halve virus quantity) infections.

**Table 3:** Example calculations for virus titration aiming for equal MOI

	Lentivirus titre (TU/ml)	Volume of lentivirus to infect 1×10^6^ cells (mL)	α:β:δ:ε_EGFP_ =1:1:1:1
α	17300000	0.058	0.058
β	12000000	0.083	0.083
δ	18400000	0.054	0.054
ε_EGFP_	8730000	0.115	0.115

***Note:*** An MOI of 1 means approximately one virion will infect a host cell, see below for example calculation. TU stands for Transducing Units.

$$\frac{TU}{mL}=\frac{No.\;cells\;at\;infection\times MOI}{mL\;of\;lentivirus\;stock}$$

At MOI=1:

$$mL\;of\;lentivirus\;stock=No.\;cells\;at\;infection\times {\left(TU/mL\right)}^{-1}$$

***Note:*** Remember to include a negative control of Expi293F cells in growth medium with polybrene but without viral infection.

5. Cover the 24-well blocks with porous adhesive film and incubate at 230–250 rpm, 37°C, 75% humidity and 8% CO_2_ for 2 days.
6. Day 3: Remove lentivirus particles by spinning down at 100–200 *g* and resuspend in Freestyle without virus or polybrene. Allow cells to recover overnight.

### Puromycin selection and expansion of infected cells

**Timing: 2 weeks**

7. Day 4: Spin down cells, and exchange media by aspirating old media and resuspending cells in 4 mL growth medium with 2.5 μg/mL puromycin.

***Note:*** For reference, cell density was around 4–5×10^5^/mL for both MOI = 1 and MOI = 0.5 samples.

8. **Day 6**: Split each well of cells into 2 wells, each with 4 mL growth medium supplemented by 5 μg/mL puromycin.

***Note:*** The MOI = 1 sample was at a density of 8.2×10^5^/mL and 85% viability, whilst MOI = 0.5 sample was at 2.1×10^6^/mL and 77% viability.

***Note:*** The exchange of medium is important to maintain antibiotic strength and to replenish volume lost to evaporation.

9. **Day 9 or 10**: Continue to exchange to fresh medium with 5 μg/mL puromycin every 1–3 days until uninfected negative control is completely dead. Discard the dead controls and begin expansion of puromycin resistant cells into larger culture volumes.
10. **Day 11–19**: Expand cells whilst maintaining a density of 0.5–2.5×10^6^/mL in 2.5 μg/mL puromycin for 1–2 weeks. Upon reaching 10^7^ total cells, freeze down 3 aliquots of 10^6^ cells for each MOI population (see step 8 of ‘before you begin’ section).

***Note:*** We decided to include additional antibiotics selection at a low concentration, but this step may not be necessary if the pre-determined duration and antibiotics concentration have been achieved (according to puromycin kill curve in preparations steps 9–12).

***Note:*** Cells may appear a bit clumpy during this stage, but this is normal.

**Pause point:** The protocol can be paused here and restarted in the future by reviving a tube of frozen cells in growth medium plus optional 2.5 μg/mL puromycin and then scaling up the cells using the above scale-up protocol.

### FACS to select quadruple-positive cells

**Timing: 2 days**

After producing a polyclonal population of puromycin-resistant cells, it is necessary to next select for clonal cell lines that express the AChR subunits using FACS.

**Figure 5:**
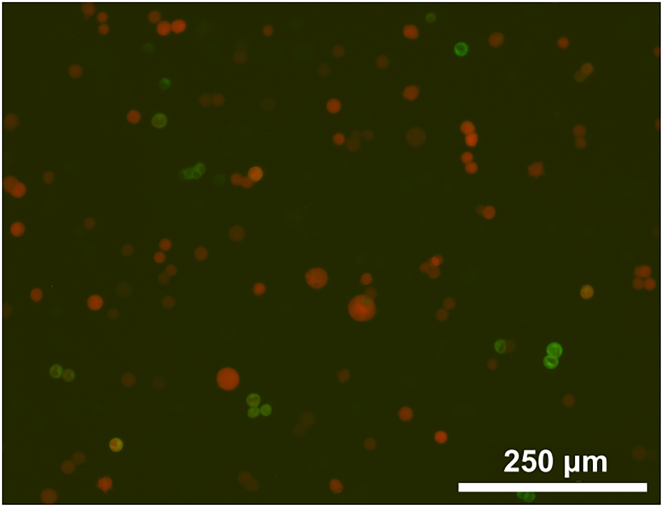
Example of EGFP and mCherry expression in cells one day post-induction for FACS Cells were infected with αβδε_EGFP_ at MOI=1.

11. **Day 20**: In a 24-well deep-well block, for each transduction MOI, seed 8 wells with 3 mL cells at 1×10^6^/mL in growth medium with 0.1 μg/mL doxycycline.
  a. Prepare the same number and volume of wells with uninfected expi293F cells as a negative control.
  b. Seal with porous adhesive film and grow overnight at 230–250 rpm, 37°C.
12. **Day 21**: One day after induction, transduced cells should be EGFP^+^ and mCherry^+^ under the fluorescence microscope (Figure 5). In a 50 mL Falcon tube, prepare **collection medium** by mixing 45 mL growth medium with 5 mL fetal calf serum (FCS).

***Optional:*** Add an additional 0.1% v/v PSA to the collection medium.

13. Prepare eighteen 96-well collection plates containing 200 μL collection medium per well.
14. Prepare 6 collection tubes with 500 μL of collection medium in 1.7 mL Eppendorf tubes for bulk culture.
15. Immediately before sorting, cells were prepared by combining the eight 3 mL cultures for each sample (MOI=1, MOI=0.5, negative control) into a 50 mL Falcon tube and centrifuging at 100–200 *g* for 3–5 min at room temperature.

**CRITICAL:** Cells should be prepared immediately before sorting to minimize time spent in ambient environment and to preserve viability.

16. Resuspend pelleted cells in 2–3 mL collection medium and transfer to a FACS tube.

***Note:*** We recommend that cells are resuspended at ∼1–3×10^7^/mL, because this high density increases the speed of sorting. Cell density may be lowered if there are concerns with high false positive rates. There was at least one false positive cell line that survived the colony expansion, as it failed to express AChR on the cell surface, and a repeat of FACS on the monoclonal cells showed it was deficient in the δ subunit.

**Figure 6:**
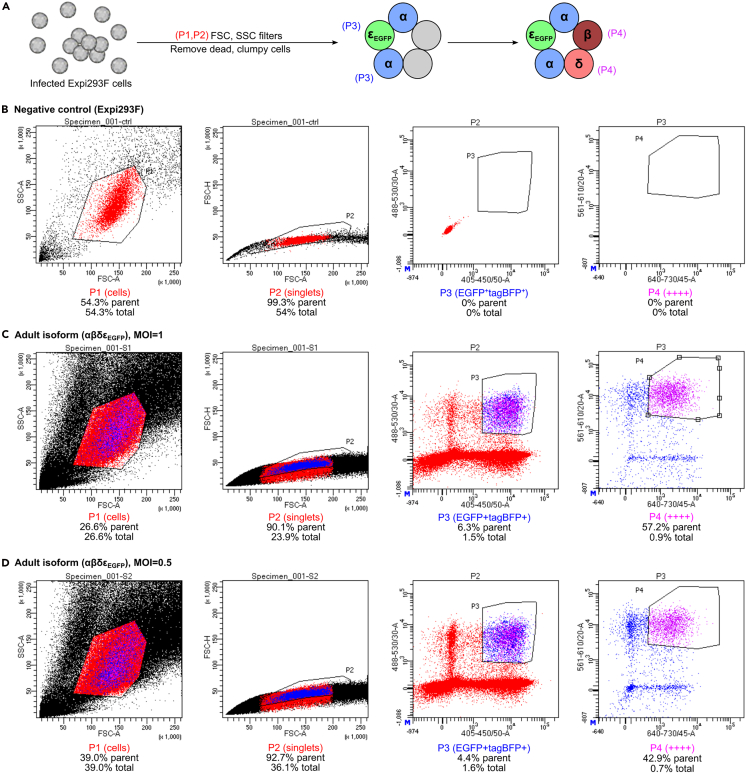
Example of FACS for αβδε_EGFP_ AChR cell line generation (A) Schematic of gating strategy. The initial FSC and SSC filters excluded cell debris and cell clumps, the tagBFP and EGFP filters selected for α^+^ε_EGFP_^+^ cells, and finally, the mCherry and mIFP filters selected for α^+^β^+^δ^+^ε_EGFP_^+^ cells (++++). (B) Uninfected Expi293F cells were sorted as negative control and contained 0% quadruple positive cells. (C and D) Infection with adult isoform virus (αβδε_EGFP_) at MOI=1:1:1:1 or 0.5:0.5:0.5:0.5 produced 0.9% and 0.7% quadruple positive cells respectively.

**Figure 7:**
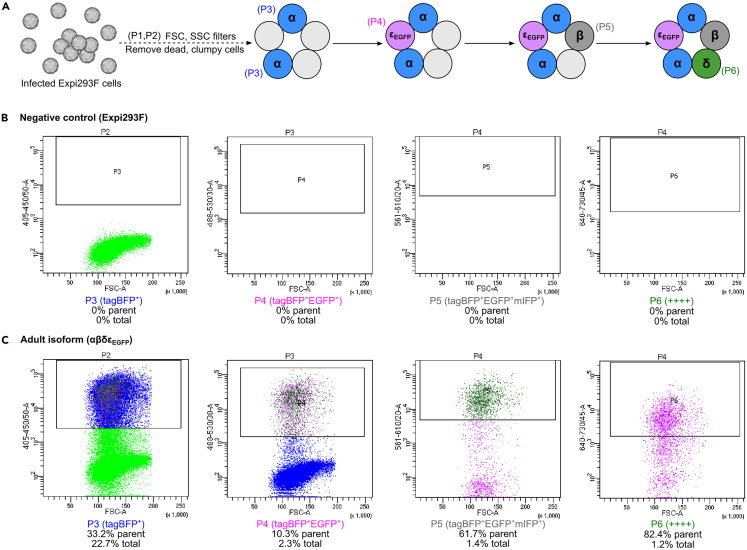
Example of FACS for αβδε_EGFP_ AChR cell line generation (A) Schematic of gating strategy. Single cells were selected with FSC and SSC filters, then gates were sequentially set for tagBFP, EGFP, mIFP and mCherry which results in α^+^β^+^δ^+^ε_EGFP_^+^ cells (++++). (B) Uninfected Expi293F cells were sorted as negative control and contained 0% quadruple positive cells. (C) Infection with adult isoform virus (αβδε_EGFP_) at MOI=1:1:1:1 produced 1.2% quadruple positive cells.
  d. Use both the negative control and infected samples to adjust the gain.
18. Sort the quadruple-positive cells at one cell per well into nine 96-well collection plates pre-aliquoted with 200 μL collection media.
19. Sort the remainder of quadruple-positive cells as polyclonal cells into the bulk collection tubes with 500 μL collection media.
20. Immediately after the sorting of each plate is complete, transfer it into a static incubator at 37°C.
21. For polyclonal cells, resuspend cells by adding growth media to a total of 3 mL, seed into a single well of a new 24-well deep-well block, and cover with porous adhesive film. Grow at 37°C, 230–250 rpm, 8% CO_2_, 75% humidity. Expand and maintain the cell lines at 0.3–3×10^6^ cells/mL under standard cell culture conditions.

***Note:*** It is possible to skip the clonal expansion and proceed straight to step 34 for protein expression test with the bulk-sorted polyclonal cells. However, this may increase the variability in expression with each cell culture.

17. Set FACS gates to sequentially select for:
  a. Cells based on Forward Scattering Area (FSC-A) and Side Scatter Area (SSC-A).
  b. Singlet cells based on scattering Forward Scattering Height (FSC-H) and Forward Scattering Area (FSC-A).
  c. Fluorochromes: tagBFP, EGFP, mCherry and mIFP (see Table 1 in ‘Before you begin’ for settings). The fluorochromes can be selected one at a time or two at a time, see Figures 6 and 7 for examples).

### Picking and expanding colonies

**Timing: 3–4 weeks**

22. Visually inspect cells in 96-well plates every ∼3 days, recording apparent density and viability. Note any wells that appear to have more than one colony and therefore might not be monoclonal.
23. **Day 28**: One week after FACS, exchange 50% of the conditioned medium with fresh growth medium, repeat every 1–5 days.

***Note:*** Fresh medium may be added to make-up for volume lost to evaporation. Alternatively, the plates may be placed in a clean open sample bag to reduce evaporation. Avoid removing the conditioned medium within the first week, which may cause cell death.

**Figure 8:**
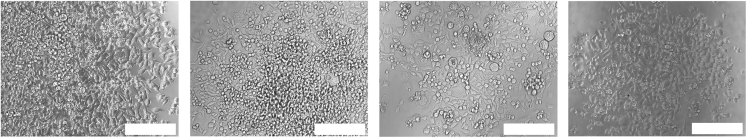
Sizes and morphologies of αβδε_EGFP_ colonies in 96-well plates, before the initial passage to 48-well plates Scale bars represent 250 μm.

***Note:*** Complete all cell washes quickly and do not allow wells to dry.

***Note:*** Passage a maximum of 5 colonies at a time to avoid mistakes or drying out.

**Figure 9:**
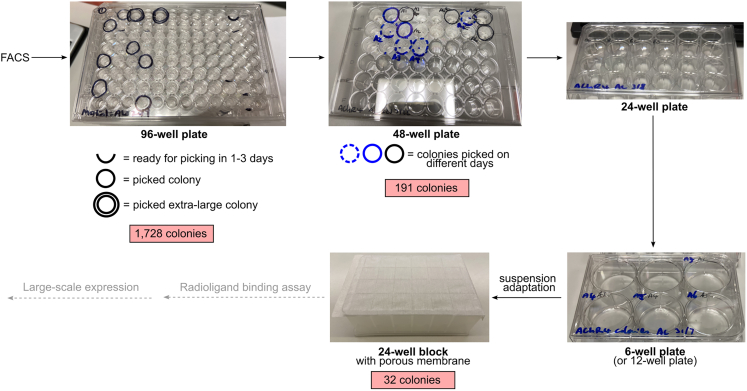
Colony expansion from 96-well plates after cell sorting to suspension adaptation in 24-well blocks The number of surviving colonies at each step is shown in pink boxes.
31. When the cells grow to ∼50–100% confluency in the 48-well plates, follow the same passage protocol (steps 24–29) and expand the cells into 24-well plates for static cell culture, and subsequently 6- or 12-well plates. Adjust the amount of PBS and trypsin-EDTA to cover the area of the well. Use 0.5–1 mL, 1–2 mL, and 1–3 mL cell culture volumes for 24-, 12- and 6-well plates.

***Note:*** The gradual expansion of colonies from 96-well plates to 48-, 24-, 12- and 6-well plates across steps 24 to 31 is key to maintaining sufficient density for cell survival.

24. When colonies expand to hundreds or thousands of cells (visible by eye without a microscope), typically about 2 weeks after cell sorting, they are ready for passaging. See examples in Figure 8.
  a. On the 96-well plate, remove medium with a vacuum aspirator.
  b. Wash the cells by addition and then aspiration of 200 μL sterile phosphate buffered saline (PBS).

25. Add 50 μL trypsin-EDTA and return the plate to the 37°C incubator until the cells dissociate, typically 3–10 min.
26. While the cells are dissociating in trypsin-EDTA, add 500 μL growth medium to sterile 1.7 mL Eppendorf tubes.
27. After the cells have dissociated, gently resuspend the cells by pipetting 3–5 times, and transfer to the prepared Eppendorf tubes. Rinse the wells once with 100–500 μL medium to get the remaining cells and transfer to the Eppendorf tubes also.
28. Centrifuge the tubes at 100–200 *g* for 3–5 min.
29. Tip out the supernatant into a Falcon tube for disposal, then resuspend the pellet in 500 μL growth medium and seed into a 48-well plate. Incubate the plate at 37°C, 5% CO_2_ in a humidified incubator.
30. Continue to harvest new colonies arising from the FACS sorted 96-well plates when these reach hundreds to thousands of cells. Clearly mark the wells that have been harvested so as not to harvest twice (Figure 9), and record any distinct colony morphology such as extra-large size.

32. Cells are ready for suspension adaptation once they reach ∼50–100% confluency in the 6- or 12-well plates.
  a. Dissociate the cells with trypsin-EDTA (steps 25–28) and seed the resuspended cells into 24-well deep-well blocks with standard growth media with 0.1–1% PSA, and cover with a sterile porous adhesive film. Grow at 37°C, 8% CO_2,_ 230–250 rpm, and 75% humidity.
  b. Subsequently, follow the standard Expi293F cell culture protocol (steps 5–7 from the Preparation section) for maintaining a density of 0.3–2.5×10^6^/mL.
33. Expand and freeze down aliquots of each cell line (see preparations section, step 8).

**Pause point:** The protocol can be paused and restarted in the future by reviving a tube of frozen monoclonal cells in growth medium according (steps 1–7 from the Preparation section).

### AChR expression test by radioligand binding assay

**Timing: 2–3 days**

This assay is adapted from an existing protocol designed for the diagnosis of AChR deficiency syndrome, where ^125^I-αBuTx is applied to intact cells to quantify cell surface AChR with correctly folded orthosteric sites.^23^ When studying plasma membrane proteins prone to intracellular retention, this method is particularly useful as it provides additional information compared to a small-scale purification involving whole-cell lysates.^34^^,^^35^

**CRITICAL:** Ensure an appropriate risk assessment is in place, and all personnel are trained for radiation work and are familiar with manufacturer’s instructions (https://resources.revvity.com/pdfs/gde-iodine125-safe-handling-guide.pdf ).

***Note:*** The half-life of ^125^I is 60.14 days, and therefore we recommended measuring the radioactivity of the stock solution before experiment. We also recommend using ^125^I-αBuTx within 4 months of delivery.

***Note:*** Ensure personal protective equipment and any additional shielding equipment is suitable for working with ^125^I. Its principal radiation emissions include gamma and X-rays. For this study, we used a lead acrylic shield between the researcher’s torso and the radiation source, and a lead acrylic-encased shaker for sample incubations.

34. Make sure there are maintenance cultures or frozen aliquots before proceeding, as the assayed cells will not be recovered for continuous culture.
  a. Seed duplicates or triplicates for cells at 0.6×10^6^ density for each cell line in 3 mL growth medium supplemented with 0.1 μg/mL doxycycline in a 24-well deep-well block covered with a porous adhesive film.
  b. Include uninfected Expi293F cells as negative control.

***Optional:*** Include additional wells for AChR-expressing cells, and negative control cells, to test if the addition of 5 mM sodium butyrate at the time of doxycycline induction to the cell culture can boost protein yield.

35. Grow cells at 37°C, 8% CO_2,_ 230–250 rpm, and 75% humidity for ∼24 h.
36. The next day, check fluorescent protein expression under a fluorescence microscope. Record the presence or absence of different fluorophores. If most cells are not yet fluorescent, return the 24-well block to the incubator for one more day of growth.
37. In the ^125^I-approved radioactivity space (hot room), measure background radiation with a hand-held gamma counter. Wipe down any area with Count-Off surface cleaner or Decon until the background radiation is below 10 counts per min (Figure 10A).

**Figure 10:**
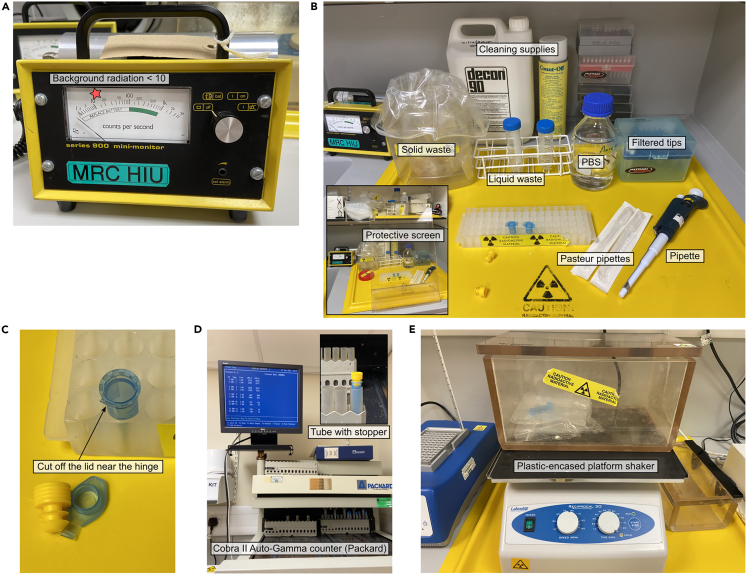
Set-up of radioligand binding assay in the hot room (A) Ensure the background radiation is <10 counts per second with a handheld gamma counter before and after experiments. (B) Set up the workbench behind a protective screen. (C and D) Cut off the lids and the protruding hinge to prevent the tube from getting stuck in gamma counters with cylindrical tube holders such as the Cobra II Auto-Gamma Counter (Packard). (E) For shaking incubation, place the tubes in a plastic bag to prevent spillage, and the bag in an encased platform shaker to shield the radiation.
38. Arrange behind a lead acrylic protective screen approved for ^125^I-emitted radiation: 50 mL Falcon tubes (for liquid waste and dilution), a plastic sample bag on holder (for solid waste), PBS (preferably sterile), disposable Pasteur pipette, tube rack, pipettes and filter tips. (Figure 10B).
39. Resuspend a 50 μCi bottle of ^125^I-αBuTx in 500 μL PBS.
40. Due to the relatively short half-life of ^125^I-αBuTx, it is good practice to measure the radioactivity of the stock and dilute to a set working concentration of 1×10^6^ cpm/mL to ensure consistency. Dilute a small amount of stock solution in PBS (e.g., 1:20), and add to an Eppendorf tube.
41. Measure the radiation inside the tube using a Cobra-II Auto Gamma Counter or similar. If using Cobra-II Auto Gamma Counter, cut off the Eppendorf tube lid and instead fit a stopper to prevent the hinge getting stuck in the counter (Figures 10C and 10D).
42. Calculate the dilution factor required to achieve 1×10^6^ cpm/mL, then dilute more ^125^I-αBuTx solution for the experiment if required.
43. In the tissue culture lab, transfer suspension cells to sterile Eppendorf tubes, pellet the cells at 100–200 g for 3–5 min and aspirate the supernatant. Record the size of cell pellets or total cell number for each cell line. Bring the cell pellets into the hot lab.
44. Add 500 μL diluted ^125^I-αBuTx per tube and gently mix by flicking. Transfer the tubes to a sealed plastic bag to prevent leakage, and incubate on a lead acrylic-encased platform shaker at ∼150 rpm for 1 hr at room temperature (Figure 10E).
45. Wash the cells 3 times by centrifuging at 4,000 g for 3 min.
  a. Discard the supernatant into liquid waste container, and resuspending in 500 μL PBS.
  b. Incubate on the platform shaker for 5 min between washes.
  c. After the last wash, spin down the cells again and remove the supernatant.
46. Measure the level of radiation using a Cobra-II Auto Gamma Counter or similar.
47. Clean up the hot lab and dispose of waste according to local regulations. Monitor background radiation to ensure it is below 10 counts per second. Fill in any necessary paperwork.

## Expected outcomes

### Analysis of AChR surface expression test

**Figure 11:**
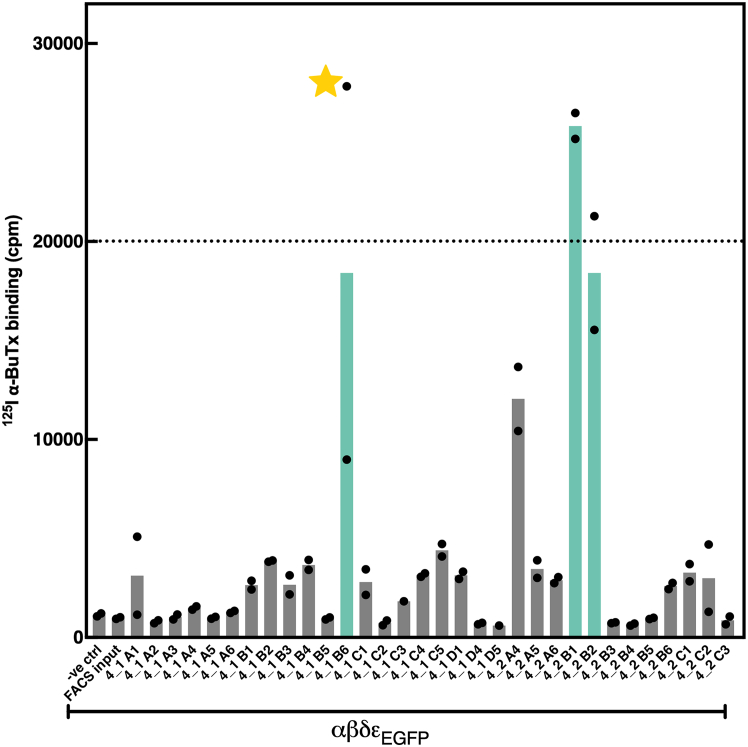
AChR surface expression measured by radioligand binding assay Negative control was uninfected Expi293F. FACS input cells had similar AChR expression levels as the negative control. Three αβδε_EGFP_-expressing cell lines were approximately 20,000 cpm to be considered suitable for large-scale protein production. Data presented are mean values (n = 2) technical repeats, except for ‘4_1 C3’ and ‘4_1 D5’ that have only a single measurement. The ‘4_1 B6’ cell line (gold star) was chosen for large-scale expression.

Based on previous ^125^I-αBuTx binding assays performed on adherent HEK293 cells transfected for transient expression of AChR, ^125^I-αBuTx labelling typically produces ∼10,000 cpm for ∼1.2×10^6^ cells (a confluent well on a 6-well plate).^23^ Thus, clones with at least two-fold higher surface AChR expression (>20,000 cpm) is deemed superior to transient transfection. Out of 32 clones, three reached the 20,000 cpm cut-off to be suitable for large-scale protein expression (Figure 11). Notably, expression should be regularly evaluated, as individual clones may lose expression due to epigenetic silencing or selection pressures. Accordingly, the ‘4_1 B6’ cell line was ultimately selected due to sustained protein expression and high viability over continuous maintenance culture of more than 4 months.

### Large-scale protein expression and purification

Expected outcomes for large scale purification can be found in the purification traces, gels, and micrographs presented in the companion paper by Li *et al.*^1^ The established cell line should yield muscle-type AChR sufficient for purification and structural or biochemical study. Membrane preparation followed by detergent solubilization, affinity purification, and size-exclusion chromatography (SEC) was carried out to purify AChR. SEC traces suggested that the AChR purified was monodisperse, and all four expected subunits were identified by tryptic-digest mass spectrometry.^1^ Proper protein folding is confirmed by an anti-AChR-α Fab fragment and αBuTx co-elution on SEC. In our structural study, the selected cell line 4_1 B6 yielded ∼6 μg per liter of cell culture. The protein is structurally homogeneous in n-dodecyl-β-D-maltoside detergent with added cholesteryl hemisuccinate by low-resolution 2D class averages, with compositional heterogeneity of the receptor assembly observed by cryo-EM.

## Limitations

While optimized for reproducible large-scale expression of AChR, this stable cell line method is not well suited for the rapid evaluation or production of new constructs or mutants due to the long time required for stable cell line selection and expansion. This can be mitigated by thorough planning in the initial construct design phase, where all anticipated constructs including mutants are cloned and processed in parallel with the wildtype. Alternatively, constructs can be pre-screened for total and cell-surface expression in Expi293F cells by transient transfection or BacMam transduction before choosing the best candidates for stable cell line generation.^34^

A potential limitation of this protocol is the multiple receptor stoichiometries that were observed when expressing adult muscle-type AChR, beyond the expected α_2_βδε. This may be a consequence of the integration for each subunit-encoding lentivirus, where the copy number and integration location are uncontrolled. While potentially informative on the requirements for the expression and assembly of multi-subunit proteins, this compositional heterogeneity can complicate biochemical and structural studies. This could potentially be mitigated by alternative strategies such as cloning the four subunits in pairs of α-δ and β-ε separated by a T2A self-cleaving peptide.^36^ Alternatively, purifying from a natural source such as beef or *Torpedo* can yield the expected stoichiometry at the expense of sequence differences from the human protein.^37^^,^^38^ Furthermore, binders such as antibodies, nanobodies, and peptide toxins can be used for sequential immunoprecipitation complexes with particular subunits or assembly interfaces, and in silico purification by 3D classification can select for particular assemblies within the cryo-EM dataset.

Finally, the lentiviral gene delivery system requires additional biosafety controls (UK BSL-2) compared to standard transient transfection, which may not be attainable for every lab. The recombinase-based Flp-In T-REx (Invitrogen), as well as transposon-based Sleeping Beauty^39^ and PiggyBac^40^ systems involve only transfection and are ready alternatives to the lentivirus approach.

## Troubleshooting

### Problem 1

Clonal expansion is at high risk of contamination (steps 22–33).

### Potential solution

• It is recommended that a mixture of antimycotic and antibiotic such as penicillin-streptomycin-amphotericin B be added at 1% v/v as opposed to the 0.1% v/v added during large-scale expression. The addition of an antimycotic is strongly recommended, as fungal contamination was observed repeatedly during this study when amphotericin B was not included in the cell culture media.
• Cell culture medium should also be replaced every 1–7 days, replacing 50% volume each time, to account for degradation of antibiotics as well as volume loss due to evaporation.
• Good sterile technique and hygiene should be practiced in the tissue culture lab, including frequent replacement of tissue-culture specific lab coat (e.g., every 1–2 weeks) and regular cleaning of the laminar flow cabinet and incubators.
• If contamination is observed in any well, it is recommended to discard the entire plate. However, if this is not possible, the affected well should be immediately decontaminated with 1% Chemgene or equivalent, and the plate monitored closely for any further contamination before passaging any remaining colonies to a new plate.

### Problem 2

Cells are all dead during clonal expansion (steps 22–33).

### Potential solution

Cell death is common when performing the initial passage of the single-cell colony on the FACS-sorted 96-well plate.

In this study, 11% of the FACS-sorted colonies survived to be transferred to 48-well plates, from which 13% colonies survived to be transferred to 24-well plates. One of the key factors to survival is cell density during seeding. A colony should reach hundreds to thousands of cells and be visible by eye before attempting passage. During this study, single-cell colonies grew at different rates and, therefore, regular evaluation of the plates over 2–4 weeks was required to recover all growing colonies.

### Problem 3

Individual target protein subunits are not expressed in the isolated cell lines (steps 34–47).

### Potential solution

The multiplicity of infection may be increased during the infection stage, for example, from 1 virion per cell (MOI=1) to 5 virus per cell (MOI=5). However, high MOI may result in increased cell death. Alternatively, this issue may be addressed by co-expressing the target with a known chaperone, such as the NACHO^41^ and TMIE^42^ known for other types of AChR. If, hypothetically, one subunit is consistently absent as indicated by its fluorescence marker, its MOI may be increased during virus titration. For example, instead of using MOI ratio of α:β:δ:ε_EGFP_=1:1:1:1, use 1:2:1:1 to boost the incorporation of β subunit.

### Problem 4

Protein expression in the stable cell line is low (steps 34–47).

### Potential solution

• Expression conditions for each mammalian protein may require optimization. These factors may include temperature (30–37°C), duration (24–72 hrs), doxycycline concentration (0.01–1 μg/mL), shaking speed (100–180 rpm), type of vessel (roller bottle vs. Erlenmeyer flask), and the timing and quantity of added sodium butyrate.
• If the protein is unstable, inhibitors, ligands or substrates added into the cell culture may sometimes act as pharmacochaperones to the protein, an example of this is ibogaine and monoamine transporters in the SLC6 family.^43^

### Problem 5

Protein expression in the stable cell line reduces over time (steps 34–47).

### Potential solution

Sometimes protein expression may be reduced due to epigenetic silencing of the transgene or selection pressure during maintenance culture.

• To address this, 5 mM of the histone deacetylase inhibitor sodium butyrate is added during protein expression.
• Cells intended for maintenance should be kept separately to avoid multiple doxycycline inductions. Thereby, each cell should be induced twice to express the target AChR protein, once before FACS and once during protein expression.
• Additionally, many aliquots of cell lines with low passage numbers should be frozen for storage. Cells should be discarded after continuous culture for 3–6 months, and an aliquot of the frozen cell line revived.

## Resource availability

### Lead contact

Further information and requests for resources and reagents should be directed to and will be fulfilled by the lead contact, Yin Yao Dong (yin.dong@ndcn.ox.ac.uk).

### Technical contact

Technical questions on executing this protocol should be directed to and will be answered by the technical contact, Anna Li (anna.li@ndcn.ox.ac.uk).

### Materials availability

All unique reagents generated in this study are available from the lead contact upon completion of a materials transfer agreement.

### Data and code availability

This protocol does not entail the use of any new datasets or codes.

## Acknowledgments

AChR subunit cDNAs were provided by Professor David Beeson from the University of Oxford. We thank Dr. Philip Hublitz and his team from the Genome Engineering Facility in the Weatherall Institute of Molecular Medicine (WIMM) for cloning the AChR lentiviral constructs and Dr. Ryan Beveridge at the Virus Screening Facility in the WIMM for generating the lentiviral particles and calculating the virus titer. We thank Craig Waugh, Dr. Paul Sopp, and Kevin Clark for assisting with cell sorting at the WIMM FACS facility. We thank Susan Maxwell (University of Oxford) for training A.L. in performing the radioactive assay. We also thank Susan Maxwell and Dr. Eleanor Williams (CMD) for kindly proofreading the manuscript. This work was supported by funding from the Wellcome Trust (102161/Z/13/Z, studentship to A.L.) and UKRI Medical Research Council (grant nos. MR/S007180/1 and MR/Z504099/1 to Y.Y.D. and MR/Y012623/1 to A.L., Y.Y.D., and D.B.S.). D.B.S. was supported by the Innovative Medicines Initiative 2 Joint Undertaking (JU) under grant agreement no. 875510 (EUbOPEN). The JU receives support from the European Union’s Horizon 2020 research and innovation program, EFPIA, the Ontario Institute for Cancer Research, the Royal Institution for the Advancement of Learning McGill University, Kungliga Tekniska Högskolan, and Diamond Light Source Limited. For the purpose of open access, the author has applied a CC BY public copyright license to any Author Accepted Manuscript version arising from this submission.

## Author contributions

Y.Y.D. and A.L. conceptualized the project. A.L. carried out the methodology and investigation. A.L. wrote the manuscript with input from D.B.S. and Y.Y.D.

## Declaration of interests

Y.Y.D. has received research funding from Argenx and Amplo Biotechnology.
